# CuInSe_2_ quantum dots grown by molecular beam epitaxy on amorphous SiO_2_ surfaces

**DOI:** 10.3762/bjnano.10.110

**Published:** 2019-05-22

**Authors:** Henrique Limborço, Pedro MP Salomé, Rodrigo Ribeiro-Andrade, Jennifer P Teixeira, Nicoleta Nicoara, Kamal Abderrafi, Joaquim P Leitão, Juan C Gonzalez, Sascha Sadewasser

**Affiliations:** 1 International Iberian Nanotechnology Laboratory, 4715-330 Braga, Portugal; 2 Departamento de Física, Universidade Federal de Minas Gerais, Belo Horizonte, 31270-901, Brazil; 3 Departamento de Física and I3N, Universidade de Aveiro, Campus Universitário de Santiago, 3810-193 Aveiro, Portugal; 4 Instituto de Microelectrónica de Madrid (CNM-CSIC), 28760 Madrid, Spain; 5 QuantaLab, 4715-330 Braga, Portugal; 6 Hassan II University of Casablanca, 20663 Casablanca, Morocco

**Keywords:** copper indium gallium selenide (CuInSe_2_), quantum dots

## Abstract

The currently most efficient polycrystalline solar cells are based on the Cu(In,Ga)Se_2_ compound as a light absorption layer. However, in view of new concepts of nanostructured solar cells, CuInSe_2_ nanostructures are of high interest. In this work, we report CuInSe_2_ nanodots grown through a vacuum-compatible co-evaporation growth process on an amorphous surface. The density, mean size, and peak optical emission energy of the nanodots can be controlled by changing the growth temperature. Scanning transmission electron microscopy measurements confirmed the crystallinity of the nanodots as well as chemical composition and structure compatible with tetragonal CuInSe_2_. Photoluminescence measurements of CdS-passivated nanodots showed that the nanodots are optoelectronically active with a broad emission extending to energies above the CuInSe_2_ bulk bandgap and in agreement with the distribution of sizes. A blue-shift of the luminescence is observed as the average size of the nanodots gets smaller, evidencing quantum confinement in all samples. By using simple quantum confinement calculations, we correlate the photoluminescence peak emission energy with the average size of the nanodots.

## Introduction

The chalcopyrite compound Cu(In,Ga)Se_2_ (CIGS) is used as the light absorber layer in thin film solar cells that typically consist of a glass substrate, a Mo back contact, the CIGS layer, and a CdS/i-ZnO/ZnO:Al front contact. The maximum power conversion efficiency achieved by this type of solar cells has recently achieved a world record of 23.35%, which outperforms all other multi-crystalline solar cells, including multi-crystalline silicon with 22.3% [1–2]. The CIGS layer in these solar cells is a thin film commonly prepared by co-evaporation in vacuum [3]. This technique consists of the simultaneous thermal evaporation of several elements under vacuum conditions and the provision of a thermal budget to the substrate [4]. Due to the excellent optoelectronic properties of this family of materials [5], new solar-cell architectures based on nanostructures have been proposed [6–9]. Examples of such architectures are intermediate-band solar cells [10], solar cells based on multi-exciton generation [11–12], quantum dot solar cells [13–15], and others [16], all with the promise to lead to significantly enhanced power conversion efficiencies beyond the Shockley–Queisser limit [17]. At this point, the research community is still developing preparation methods for such nanostructures. Several works have demonstrated the growth of CuInSe_2_ (CIS) nanodots using solution-based processes [18–26]. However, for thin film solar cells prepared by non-vacuum methods the resulting devices usually yield a significantly lower electrical performance compared with vacuum-prepared solar cells [27–28]. This difference between the electrical performance of vacuum-prepared materials and non-vacuum-prepared materials is usually attributed to the sensitivity of the chalcopyrite semiconductor to external contaminants [29], to the dependency of its properties on the preparation method [30], and to the self-doping characteristics of the chalcopyrite materials [31].

The motivation behind the present work is to grow CIS nanodots on substrates covered with an amorphous layer using co-evaporation. The vacuum conditions of the co-evaporation process will allow for the CIS to keep adequate optoelectronic properties, as demonstrated by the photoluminescence analysis. The use of a substrate with an amorphous surface, together with the use of an industrial standard growth technique, namely co-evaporation, opens the door to use the nanodots in advanced solar-cell architectures and a potential large-area industrialization of this process.

## Experimental

### Sample preparation

The samples presented in this work were grown in a molecular beam epitaxy system (Omicron EVO 50) by evaporating high-purity solid precursors. Nanodots were grown on Si(100) with an approximately 1.6 nm thick layer of native SiO_2_. The substrates were outgassed at 600 °C for 10 min before the growth. The background pressure in the reaction chamber was monitored during the entire deposition by a hot-filament ion gauge. Just before the start of the deposition the pressure was of the order of 10^−8^ mbar and after opening the effusion-cell shutters it rapidly increased to about 10^−7^ mbar, to which it stabilized until the end of the process. Conventional hot-lip Knudsen cells were used as Cu and In sources and a valved selenium cracking source was used to evaporate Se. Evaporation fluxes of 1.4 × 10^13^ atoms·cm^−2^·s^−1^ and 2.6 × 10^13^ atoms·cm^−2^·s^−1^ were used for Cu and In, respectively. Se was evaporated in excess of 30 times, as it is typical for chalcopyrite materials [32–34]. All samples presented in this work were deposited for a period of 5 min and with a substrate rotation of 10 rpm, to improve the homogeneity of the samples. Under these conditions a nominal CIS growth rate of 6 ± 1 Å/min was determined by measuring the thickness of thick CIS calibration samples. To avoid Se loss, the samples were cooled down from the maximum growth temperature to 300 °C at 10 °C/min under Se flux. Below 300 °C, the cooling rate was reduced to approximately 5 °C/min and the Se flux was ceased. In this work we present samples prepared at substrate temperatures of 490, 530, and 580 °C; they will be referred to by their growth temperature.

### Characterization

The surface morphology was determined by scanning electron microscopy (SEM) in a FEI Quanta 650 FEG SEM microscope. Scanning transmission electron microscopy (STEM) images were taken with a FEI Titan ChemiSTEM 80-200 kV Cs-probe corrected transmission electron microscope, operating at 200 kV accelerating potential and equipped with an energy-dispersive X-ray spectroscopy (EDS) SuperX-Bruker silicon drift detector. In this method a coherent focused probe scans across the specimen and the X-ray emission spectrum is recorded in each probe position. To completely discard the possibility of EDS superposition problems, for the linescan measurements we checked both L- and K-emission lines of Cu, In, and Se; however, for simplicity we will only present one line for each element. The STEM lamellae were prepared in a focused ion beam (FIB) FEI Dual-Beam Helios 450S with FIB Mo-grids using a technique known as “lift-out” [35]. To improve FIB preparation and visualization of the nanodots in the STEM, the samples were coated with an amorphous carbon layer prior to the protective Pt bi-layer deposition assisted by electron and ion beams. The final polishing of the lamella was done using 1 keV in energy to reduce the lateral damage and the Ga implantation effects in the lamellae. EDS was performed in the same STEM microscope in order to check the [Cu]/[In] ratio of the samples, and to validate the oxidized nature of the substrate surface.

Photoluminescence (PL) experiments were carried out with Bruker IFS 66v Fourier-transform infrared (FTIR) spectrometer, equipped with a liquid nitrogen cooled Ge diode detector for which the spectral range of detection is 0.70 to 1.55 eV. The excitation source was the 514.5 nm line of an Ar^+^ ion laser (spot diameter of ca. 1 mm). PL spectra were taken at 7 K with an excitation power between 104 and 220 mW. It was not possible to observe a photoluminescence emission band for the as grown samples, thus a CdS passivation layer was deposited on top of the samples. CdS was deposited by conventional chemical bath deposition (CBD) with a solution of 1.1 M ammonia, 0.100 M thiourea, and 0.003 M cadmium acetate [36]. The solution is mixed in a beaker at room temperature, and the samples are immersed into the beaker, which is subsequently heated to 60 °C in a water bath. During the growth process, the solution is stirred for 10 s each minute. The duration of the bath was 9 min, and the samples are then directly removed from the CBD beaker and immersed in clean deionized water to stop the growth process. This process typically produces films with a thickness of 50–70 nm.

## Results and Discussion

**Figure 1:**
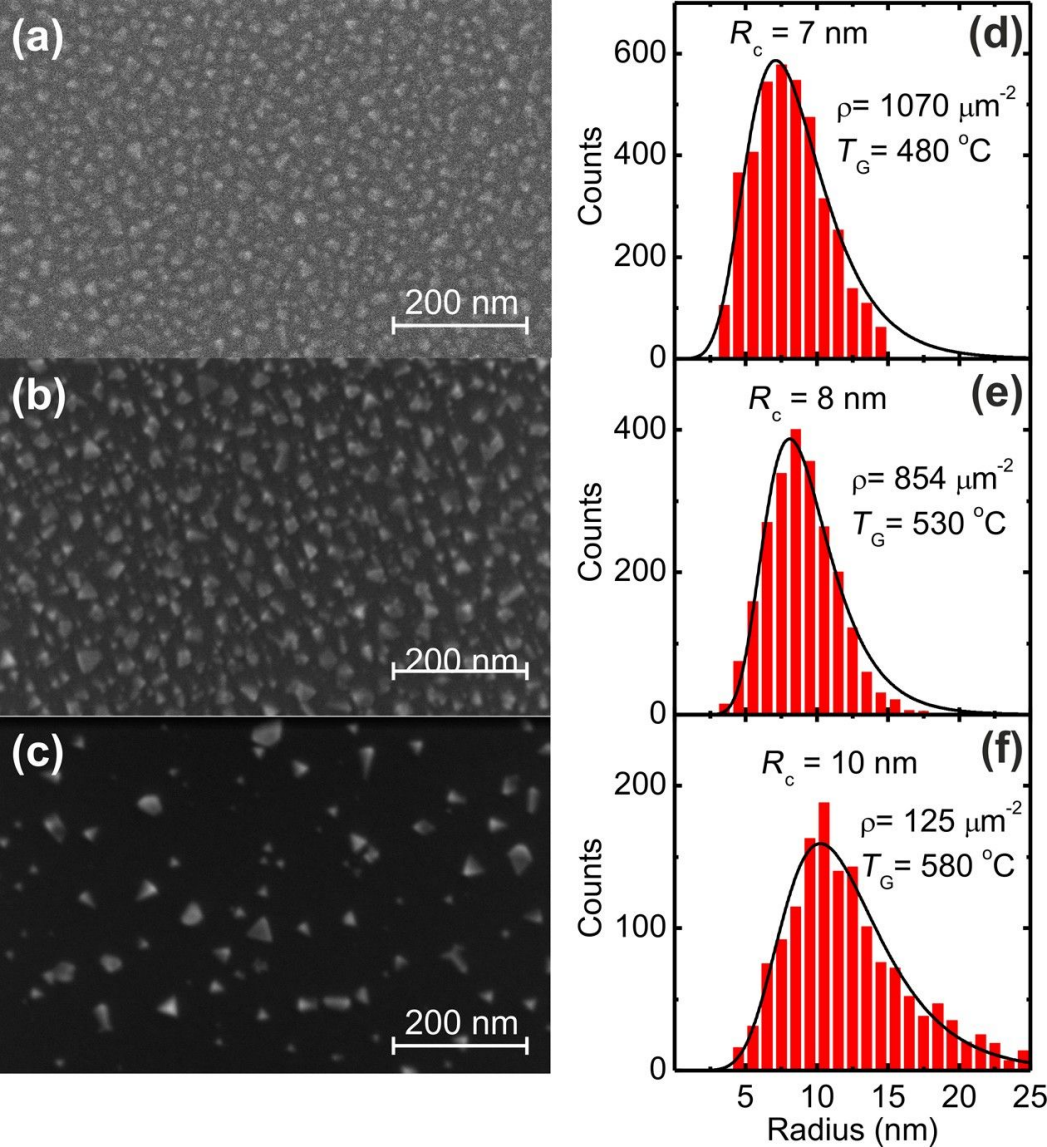
Representative SEM images of samples grown at (a) 490 °C, (b) 530 °C, and (c) 580 °C, showing a large surface density of triangular nanodots. Lateral size histograms of the nanodots grown at (d) 490 °C, (e) 530 °C and (f) 580 °C. *R*_c_ corresponds to the most probable value of the base radius of the nanodots, while ρ is the areal density of the nanodots.

We prepared three samples grown on Si(100) substrates with a ca. 1.6 nm layer of native SiO_2_ using exactly the same evaporation fluxes but at different substrate growth temperatures (*T*_G_) of 490, 530, and 580 °C. Figure 1 shows representative scanning electron microscopy (SEM) images of each sample surface. At the lowest growth temperature, 490 °C, Figure 1a, there is already evidence that individualized and separated nanodots are formed on the surface. All samples presented a large areal density of crystalline nanodots with a predominant pyramidal shape (as confirmed by SEM and STEM images). With increasing temperature the nanodots increase in size and are more separated, Figure 1b,c. Additional SEM images of all samples were analysed for the evaluation of lateral sizes and areal densities of the nanodots. The corresponding CIS nanodot size histograms are shown in Figure 1d–f. They show broad and asymmetric distributions of nanodots. The nanodots average size *R*_c_ (defined by the average equivalent base area radius of the nanodots) slightly increases with the growth temperature, while the areal density ρ of the nanodots decreases almost one order of magnitude. It is clear that with increasing growth temperature coarsening and In re-evaporation might play important roles in the nanodots size distribution.

**Figure 2:**
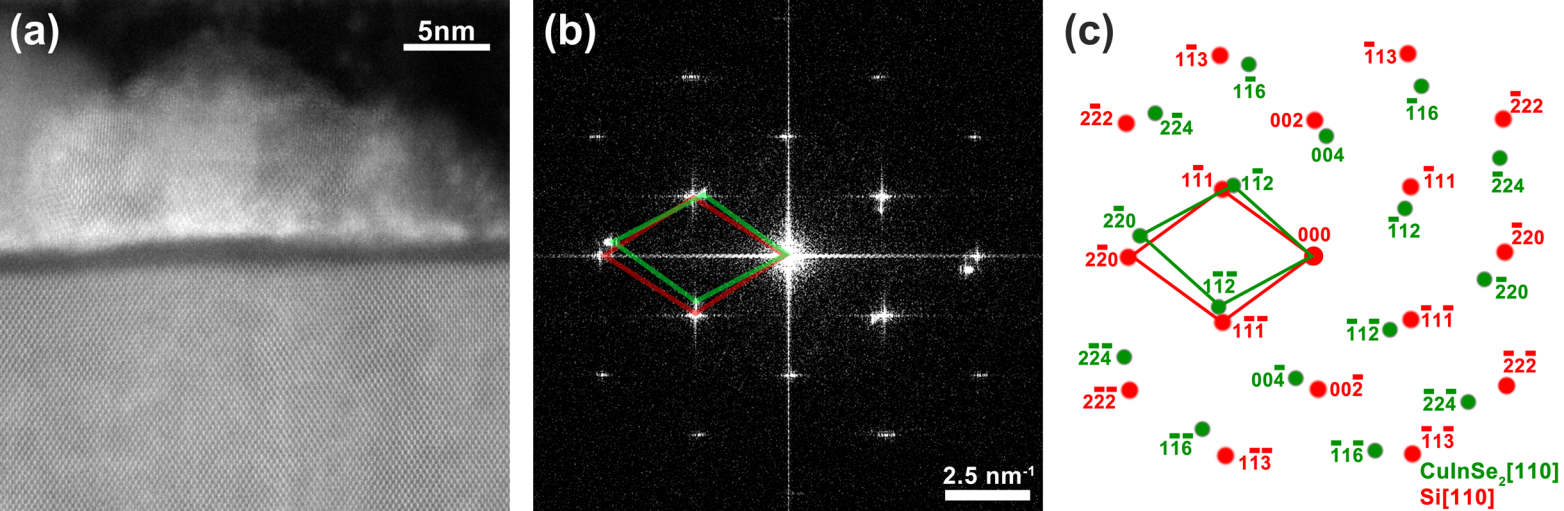
(a) High-resolution HAADF image of a nanodot on top of the amorphous SiO_2_ layer, taken along the [110] zone axis of the Si substrate. (b) Power spectrum of the HAADF image, showing the nanodot and Si substrate lattices. (c) Extracted pattern showing the indexed diffraction spots of the nanodot (green) and Si substrate (red).

The crystal structure of the nanodots was determined by scanning transmission electron microscopy (STEM) analysis performed on the 530 °C sample. Figure 2a shows a high-resolution cross-section high-angle annular dark-field (HAADF) image of a nanodot. Due to the large density of nanodots in the sample, a superposition of two other nanodots can be observed on the back (left and right sides of the image) of the nanodot under study. The approximately 1.6 ± 0.3 nm thick amorphous SiO_2_ layer is also observed, isolating the nanodot from the Si substrate. A power spectrum (PS) generated from the HAADF image is presented in Figure 2b. Figure 2c presents the simulated diffraction patterns along the [110] zone axis for Si and tetragonal CIS [37]. Interplanar distances of 0.322, 0.185, and 0.322 nm were found from the nanodot diffraction spots (−112), (−220), and (−11−2), respectively. From the reference X-ray diffraction (XRD) database, the interplanar distances of (−112), (−220), and (−11−2) are 0.335, 0.205, and 0.335 nm, respectively [31]. The interplanar distances as well as the nanodot PS are in agreement with the tetragonal structure of CuInSe_2_. It is important to point out that epitaxial relationships are not expected between the nanodots and the Si substrates, since the CIS nanostructures are actually grown on top of an amorphous layer as evidenced by the STEM analysis.

**Figure 3:**
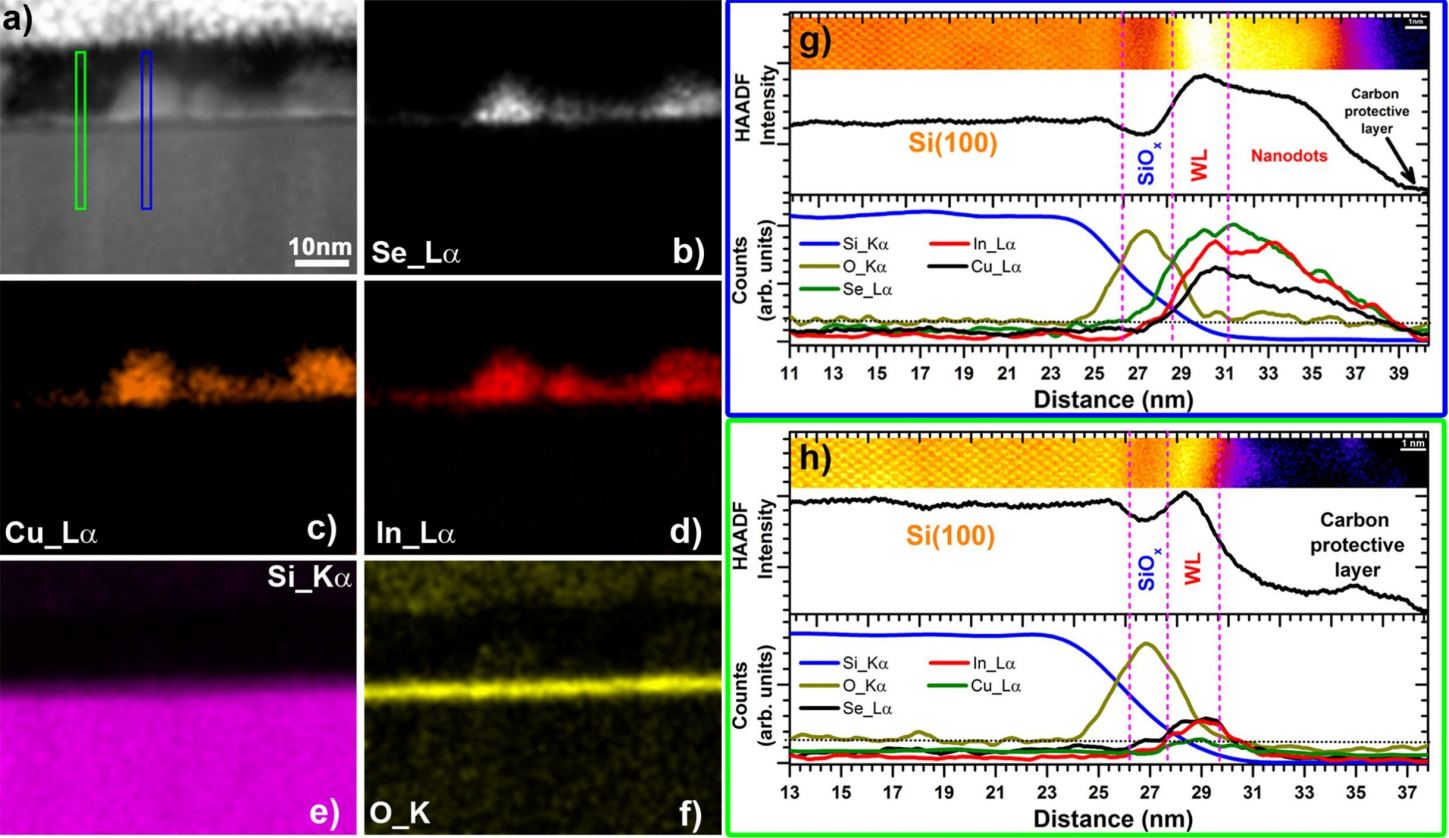
Chemical analysis of the sample grown at 530 °C. (a) Low-resolution cross-section STEM HAADF image of a region of the 530 °C sample studied by EDS. (b–f) EDS mapping of Se, Cu, In, Si, and O. (g) EDS line profile across a nanodot (blue rectangle in (a)). (h) EDS line profile across the WL (green rectangle in (a)).

Energy-dispersive X-ray spectroscopy (EDS) mapping was carried out in the 530 °C sample in order to determine the chemical composition of the nanodots. Figure 3a–f, shows a low-resolution cross-section HAADF image of the sample, as well as the EDS chemical maps of that area for Se, Cu, In, Si, and O. The EDS mapping confirms that the nanodots are grown on top of a SiO_2_ layer, as shown by the presence of an oxygen-containing layer between the dots and the Si substrate. An EDS quantification performed in this nanodot (and several others) confirmed the chemical composition of [Cu]/[In] = 1.05 ± 0.13 and [Se]/[metal] = 0.83 ± 0.14 (average value ± standard deviation). These values are compatible with a CuInSe_2_ chemical composition of the nanodots and are also in agreement with the structural characterization presented above. Two line profiles of a region with a nanodot (blue rectangle in the HAADF image) and a region without a nanodot (green rectangle in the HAADF image) were extracted from the chemical maps and are presented in Figure 3g and Figure 3h, respectively. For comparison, a section of the HAADF images as well as intensity contrast along the rectangular areas are also shown on the top panels. The HAADF images combined with the EDS line profiles reveal a 1.3 ± 0.3 nm thick layer formed in the region in-between nanodots with bright contrast and a [Cu]/[In] = 0.6 ± 0.3 and [Se]/[metal] = 1.2 ± 0.3 chemical composition. This composition suggest the formation of a Cu–In–Se ordered defect compound, which has been reported to form along the tie-line of the (Cu_2_Se)*_x_*–(In_2_Se_3_)_1−_*_x_* pseudo-binary system [38–39], and which has a bandgap energy very close to that of CuInSe_2_ [40]. In this compositional region, i.e., low amounts of Cu compared with In, the so called Cu-poor CIS composition, CIS is capable of maintaining its crystalline structure as well as its optoelectronic properties [39]. We interpret this thin layer as a 2D wetting layer (WL) formed during the first steps of growth that transits to 3D nanodots as the growth continues. This WL also appears as a non-uniform thin and bright layer, on top of the SiO_2_ layer and below the nanodots, across the whole HAADF image in Figure 3a. However, due to the difficulties in doing reliable chemical and structural measurements in such a thin and inhomogeneous layer further research is necessary in order to understand the structure, chemical composition, and formation of this thin layer, as well as the possible 2D–3D transition responsible for the formation of the nanodots.

**Figure 4:**
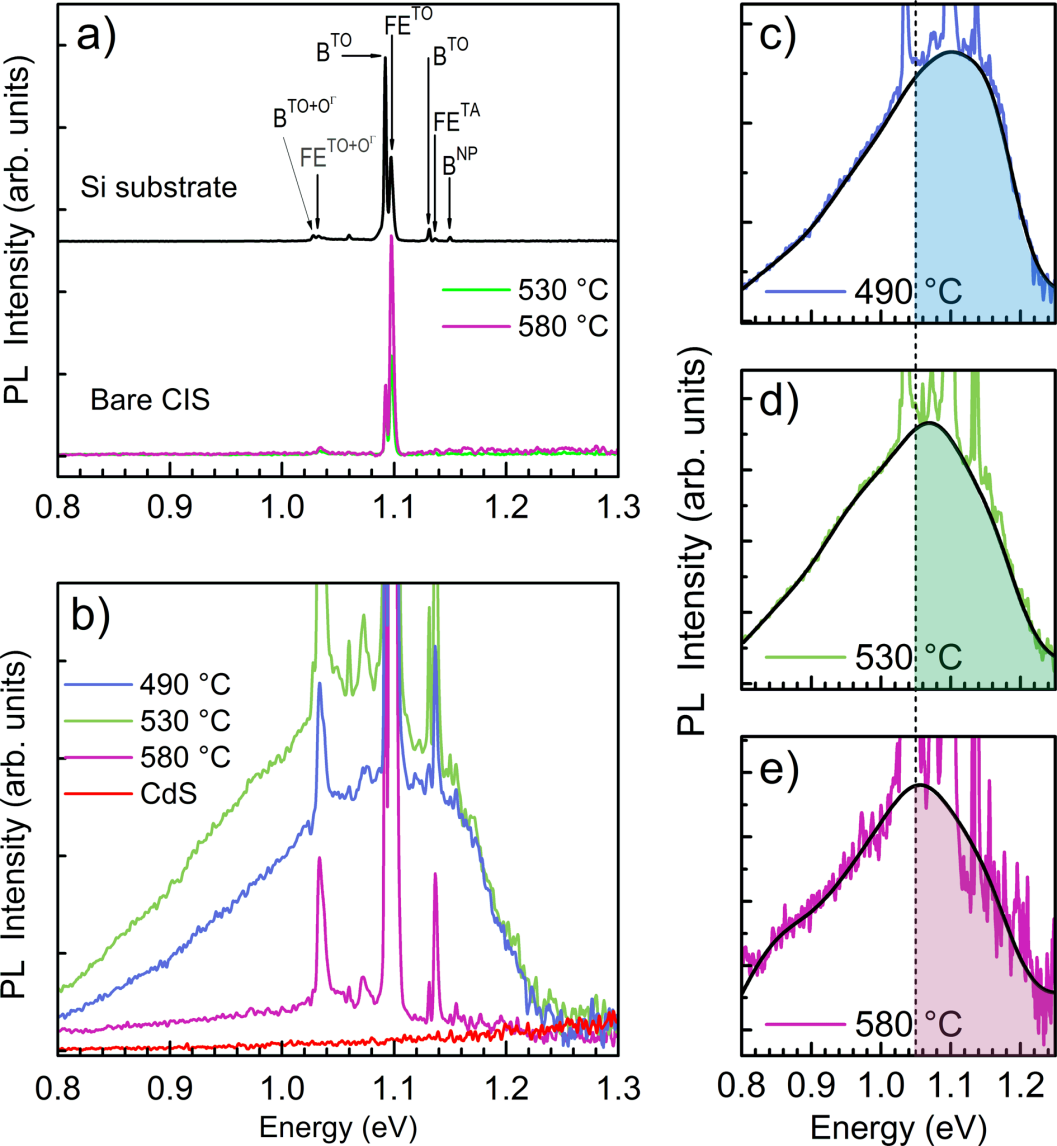
(a) PL spectra measured at 7 K and with an excitation power of 104 mW of a Si substrate and the bare CIS nanodots samples. (b) PL Spectra of the same CIS samples (in (a)) covered with CdS, measured at the same temperature and with an excitation power of 220 mW. A PL spectrum of a CdS layer deposited on top of a glass covered with a Mo layer is shown for comparison. (c), (d), and (e) show, for each sample, the broad band CIS-related emission superimposed on the Si-related peaks. The dashed line indicates the bulk bandgap of CIS at low temperature.

In order to study the optoelectronic properties of the CIS nanodots, photoluminescence (PL) measurements were performed on bare Si substrates, bare (as-grown) nanodot samples and CdS-covered nanodot samples, as shown in Figure 4. The PL spectra of the bare Si and CIS samples, Figure 4a, show only the common sharp lines related with the Si substrate [41–42]. The observed lines correspond to the phonon replicas (TA, TO and TO+OΓ) of the radiative recombination of free excitons (FE) and of bound excitons (B), as well as the no-phonon line of the bound exciton (B^NP^) as confirmed from PL measurements performed under the same experimental conditions of a Si substrate.

After the deposition of a CdS layer on top of the three as-grown nanodot samples, new PL measurements were performed under exactly the same experimental conditions, shown in Figure 4b. A broad band between 0.8 and 1.2 eV is observed for all three samples, which is superimposed on the sharp lines related to the Si substrate discussed previously. To evaluate the possible influence of the CdS layer on the measured luminescence, a PL spectrum of a CdS layer deposited on glass covered with a Mo layer, measured under the same experimental conditions, is also shown in Figure 4b. No significant luminescence related with the CdS layer was observed. Intrinsic defects and surface defects are important sources of radiative and non-radiative recombination affecting the optoelectronic properties of CIS quantum dots (QDs), such as photo-generated carrier lifetime and photoluminescence [15]. We ascribe the increase in the photoluminescence related to the nanodots to the passivation of surface states in the nanodots by the CdS deposition process. The passivation of Cu(In,Ga)Se_2_ surfaces by a CdS layer is well known [38] and passivation of nanodots is known to increase their PL signal [43–44].

In order to facilitate a comparison of the broad band emission between the three samples, we show in detail the spectra in Figure 4c–e. The dashed line indicates the bulk bandgap of CIS (1.04–1.05 eV) [45–46]. It is clear that all samples show peak emission at energies above the bulk bandgap, which we preliminarily explain by a quantum confinement effect due to the small mean size of the nanodots presented in Figure 1. With increasing substrate temperature, the mean size of the nanodots increases, leading to reduced quantum confinement effect and a red-shift of the PL emission to values closer to the CIS bandgap.

To analyse the CIS emission in more detail and independently of the superposition with the sharp Si emissions from the substrate, we used several Gaussian components to fit the CIS-related emission (solid black lines). We note that no physical meaning can be ascribed to these Gaussian components, they merely serve to estimate the peak energy of each broad luminescence spectrum. For the 490 °C sample we estimate a maximum peak emission at 1.10 ± 0.02 eV, for 530 °C at 1.069 ± 0.015 eV, and for 580 °C at 1.05 ± 0.02 eV. We note that the spectral resolution of the PL system is well below the given uncertainties, which mostly result from the fitting uncertainty. The determined peak energies confirm the qualitatively observed red-shift of the PL emission with increasing average size of the nanodots resulting from the increased growth temperature. We note here that a potential re-evaporation of In at higher growth temperatures might also affect the stoichiometry leading to a higher Cu-to-In ratio. However, it has been shown that the bandgap of CIGS exhibits a blue-shift with decreasing Cu-to-In ratio [47], which is opposite to our observation. Therefore, we can exclude that the observed change in bandgap is of stoichiometric origin.

In addition to the nanodots, PL emission could also be expected from the Cu-poor CIS wetting layer. We would expect the WL to behave as a thin (ca. 1.3 nm) quantum well with a narrow PL emission. However, the TEM analysis is highly localized, and the thickness of the layer could vary in other regions of the sample. To analyse the PL emissions in more detail, simple quantum confinement calculations were carried out considering a hard wall spherical QD of radius *R* and the wetting layer as a hard wall quantum well (QW) of thickness *L*. The calculations consider the free exciton emission for the QD and QW, respectively. The first transition energy for the QD [48] and QW [49] can be calculated as:

[1] $$E_{\text{QD}}=E_{\text{g}}+\frac{h^{2}}{8R^{2}}\left(\frac{1}{m_{\text{e}}}+\frac{1}{m_{\text{h}}}\right)-\frac{1.786e^{2}}{4\pi \varepsilon R},$$

[2] $$E_{\text{QW}}=E_{\text{g}}+\frac{h^{2}}{8L^{2}}\left(\frac{1}{m_{\text{e}}}+\frac{1}{m_{\text{h}}}\right)-E_{\text{x}},$$

where *E*_g_ is the CIS low-temperature bandgap energy, *h* is the Planck constant, *m*_e_ is the effective conduction-band mass, *m*_h_ is the effective valence-band mass, *e* is the rest electron charge, ε is the CIS dielectric constant and *E*_x_ is the exciton binding energy in the QW [50].

**Figure 5:**
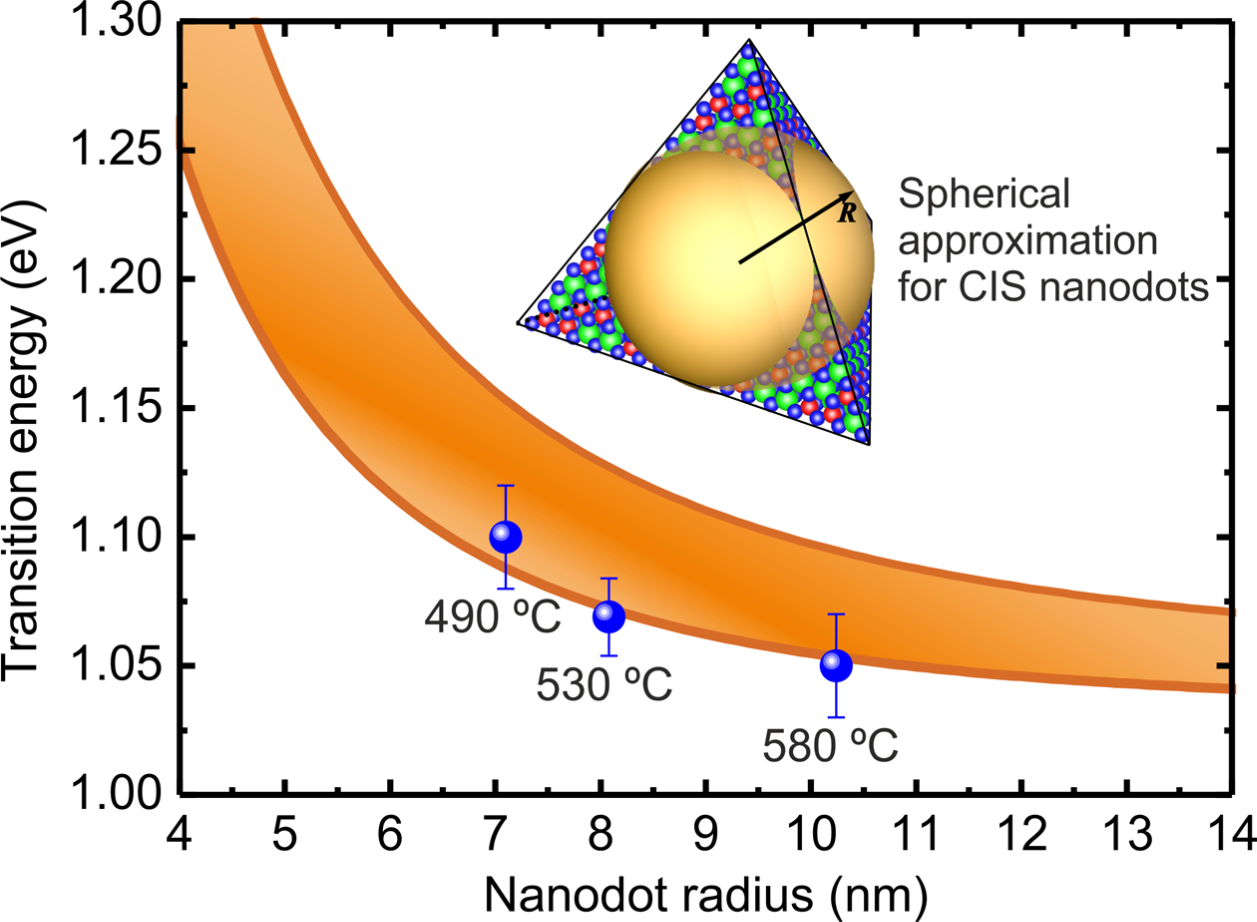
Simulated transition energy as a function of the size of CIS nanodots according to Equation 1. The illustrated range of transition energies is due to the variation of literature values of the parameters of Equation 1. The experimentally determined PL emission peak energy of the nanodots samples is represented by solid blue data points.

To compare the experimental data with predictions from the simple QD confinement simulations, Figure 5 shows the first (lowest energy) transition energy as a function of *R* for the QD (for simplicity, we approximate the QD shape as spherical, as indicated in the inset to Figure 5, since the exact shape is unknown). The figure shows a band of transition energies corresponding to the spread of values found in the literature for the parameters in Equation 1: *E*_g_ = 1.04–1.05 eV [45–46], *m*_e_ = 0.09*m*_0_ (where *m*_0_ is the electron rest mass) [51], *m*_h_ = 0.23–0.73*m*_0_ [52–55], and ε = 9.3–16ε_0_ (where ε_0_ is the vacuum electrical permittivity) [52,55–56]. The experimentally determined peak emission energies are also plotted, as a function of *R*_c_ for each sample. Clearly, the obtained values are in agreement with the emission from the quantum-confined energy levels in CIS nanodots.

According to Equation 2, the expected transition energy for the QW is in the range of 3.77 to 4.78 eV for the observed 1.3 nm thick WL, and drops to 2.18 to 2.49 eV for a 2 nm thick QW. The values of the parameters and the spread for the QW calculations were considered to be equal to the CuInSe_2_ parameters for the QD. These transition energies are significantly above the observed PL broad emission of the CdS-passivated nanodots samples and cannot be actually observed in our PL measurements due to the spectral range of detector and optical filters used. Further investigations with a more realistic model to simulate the PL emission from these types of structures and deeper optical characterization of this kind of sample is needed to better understand the optical properties for both the WL and the nanodots.

Despite of the arguments (very small mean size of the CIS QDs, increasing mean size of the CIS QDs with growth temperature, blue-shifted PL peak, and quantum confinement calculations in agreement with the blue-shifted PL peak), further investigations are needed to fully understand the quantum confinement in these nanostructures.

## Conclusion

CIS nanodots were grown on substrates with an amorphous SiO_2_ surface. By varying the growth temperature, it was possible to control the nanodots mean size, areal density, and peak emission energy. It is the first time that CIS nanodots were prepared in a single step under vacuum conditions on an amorphous substrate. STEM and EDS analyses reveal a crystal structure and chemical composition of the dots compatible with the CIS tetragonal structure and stoichiometry. The same analysis also hints to a Cu-poor and Se-rich CIS wetting layer, but the effects, structure, and exact composition of this layer have to be studied in more detailed. PL measurements of the three samples show a broad emission, peaking at energies above the CIS bandgap, and resembling the nanodots size distribution. For the samples prepared at the lower temperature, which provides the smallest nanodots, the PL emission is blue-shifted compared with the sample grown at the highest temperature, which has the biggest nanodots. A simple quantum confinement model used to calculate the first transition energy of the nanodots shows a good agreement between the average size of the dots and the observed peak emission energy. The correlations between the size distribution of the nanodots and the PL emission band together with the quantum confinement model indicate that the observed PL emission originates from quantum-confined states in the CIS nanodots. An additional important observation of the PL analysis is the evidence of the surface passivation of the nanodots by a wet-deposited thin CdS layer, allowing the nanodots to be optoelectronically active. The fabrication of these nanostructures in a vacuum environment and on an amorphous substrate is very interesting from an industrial point of view for photovoltaic applications.
